# Percutaneous Endoscopic Gastrostomy, Duodenostomy and Jejunostomy

**DOI:** 10.1155/DTE.1.37

**Published:** 1994

**Authors:** Yukio Nishiguchi, Yuichi Fuyuhiro, Jae-To Lee, Soon-Myoung Kang, Mitsuru Baba, Yuichi Arimoto, Kazuhiro Takeuchi, Yoshito Yamashita, Akira Shigesawa, Kazuhiko Yoshikawa, Michio Sowa

**Affiliations:** 1 First Department of Surgery Osaka City University Medical School Osaka Japan; 2 Department of Surgery Baba Memorial Hospital Japan; 3 First Department of Surgery Osaka City University Medical School, 1-5-7 Asahi-machi Abeno-ku Osaka city Osaka 545 Japan

## Abstract

Although enteral feeding by nasal gastric tube is popular for the patients who have a swallowing disability and require long-term nutritional support, but have intact gut, this tube sometimes causes aspiration pneumonia or esophageal ulcer. For these patients, conventional techniques for performance of a feeding gastrostomy made by surgical laparotomy have been used so far. However, these patients are frequently poor anesthetic and operative risks. Percutaneous endoscopic gastrostomy (PEG) which can be accomplished with local anesthesia and without the necessity for laparotomy has become popular in the clinical treatment for these patients. PEG was performed in 31 cases, percutaneous endoscopic duodenostomy (PED) in 1 case, and percutaneous endoscopic jejunostomy (PEJ) in 2 cases. All patients were successfully placed, and no major complication and few minor complications (9%) were experienced in this procedure. After this procedure, some patients could discharge their sputa easily and their pneumonia subsided. PED and PEJ for the patients who had previously received gastrostomy could also be done successfully with great care. Our experience suggests that PEG, PED, and PEJ are rapid, safe, and useful procedures for the patients who have poor anesthetic or poor operative risks.


*Diagnostic and Therapeutic Endoscopy*, Vol. 1, pp. 37–43
Reprints available directly from the publisher
Photocopying permitted by license only

© 1994 Harwood Academic Publishers GmbH
Printed in Malaysia

### Percutaneous Endoscopic Gastrostomy, Duodenostomy and Jejunostomy

YUKIO NISHIGUCHI*, YUICHI FUYUHIRO**, JAE-TO LEE**, SOON-MYOUNG KANG**, MITSURU BABA**, YUICHI ARIMOTO*, KAZUHIRO TAKEUCHI*, YOSHITO YAMASHITA*, AKIRA SHIGESAWA*, KAZUHIKO YOSHIKAWA* and MICHIO SOWA*

**First Department of Surgery, Osaka City University Medical School, Osaka, Japan*
***Department of Surgery, Baba Memorial Hospital*

*(Received October 28, 1993; in final form January 6, 1994)*

Although enteral feeding by nasal gastric tube is popular for the patients who have a swallowing disability and require long-term nutritional support, but have intact gut, this tube sometimes causes aspiration pneumonia or esophageal ulcer. For these patients, conventional techniques for performance of a feeding gastrostomy made by surgical laparotomy have been used so far. However, these patients are frequently poor anesthetic and operative risks. Percutaneous endoscopic gastrostomy (PEG) which can be accomplished with local anesthesia and without the necessity for laparotomy has become popular in the clinical treatment for these patients. PEG was performed in 31 cases, percutaneous endoscopic duodenostomy (PED) in 1 case, and percutaneous endoscopic jejunostomy (PEJ) in 2 cases. All patients were successfully placed, and no major complication and few minor complications (9%) were experienced in this procedure. After this procedure, some patients could discharge their sputa easily and their pneumonia subsided. PED and PEJ for the patients who had previously received gastrostomy could also be done successfully with great care. Our experience suggests that PEG, PED, and PEJ are rapid, safe, and useful procedures for the patients who have poor anesthetic or poor operative risks.

**KEY WORDS:** endoscopic gastrostomy

### INTRODUCTION

Patients unable to take oral alimentation require enteral feeding by nasogastric tubes. These tubes have been shown to cause many complications such as esophagitis, gastroesophageal reflux, and aspiration pneumonia (Yamada *et al.*, 1991). Therefore, such tubes are not suitable for long-term alimentation. For these patients, surgical gastrostomy were being made by surgical laparotomy. These patients are frequently poor anesthetic and operative risks. For these patients, percutaneous endoscopic gastrostomy (PEG) has become an accepted procedure. Our experience with PEG, including procedure and complications is reported.

Address for correspondence: Yukio Nishiguchi, First Department of Surgery, Osaka City University Medical School, 1-5-7, Asahi-machi, Abeno-ku, Osaka City, Osaka 545, Japan.

### MATERIALS AND METHODS

After careful determination that no other method of alimentation was superior, patients were selected for PEG. The Sacks-Vine Gastrostomy Kit (Dainabott Co., Ltd., Japan) was used for this procedure (Fig. 1). We followed the procedure reported by Okano (Okano *et al.*, 1986) (Fig. 2). With the endoscope in place in the stomach, the patient was rolled into the supine position and the stomach insufflated with air. With the room lights dimmed, the endoscope is deflected to the anterior surface of the Seldinger needle. This site is usually 1/3 the distance from the left costal margin at the midclavicular line to the umbilicus. The insertion site is depressed with a finger (Fig. 3a). The endoscopist should clearly see the resulting depression on the anterior surface of the gastric wall. Using a local anesthesia, a small incision in the abdominal wall was made (Fig. 3b). The Seldinger needle was inserted

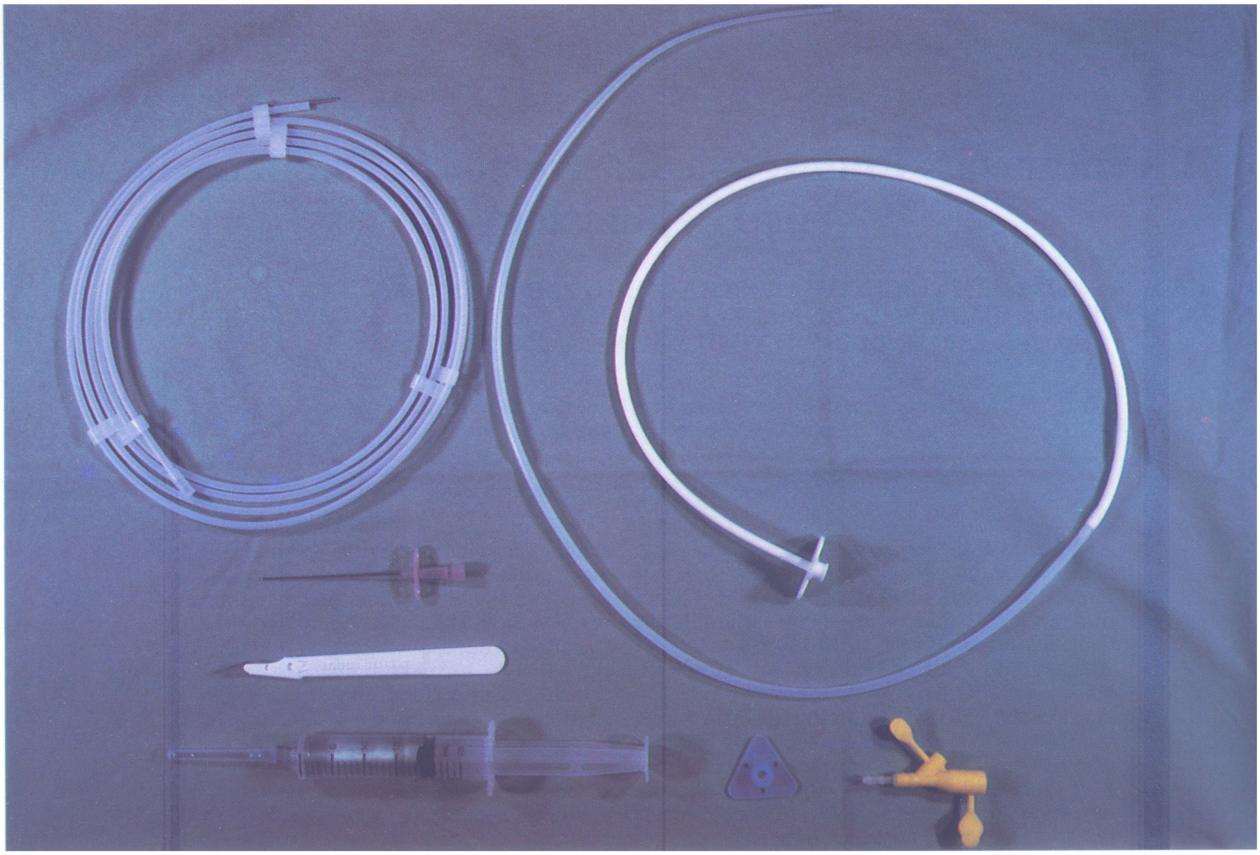

**Figure 1** Sacks-Vine Gastrostomy Kit.

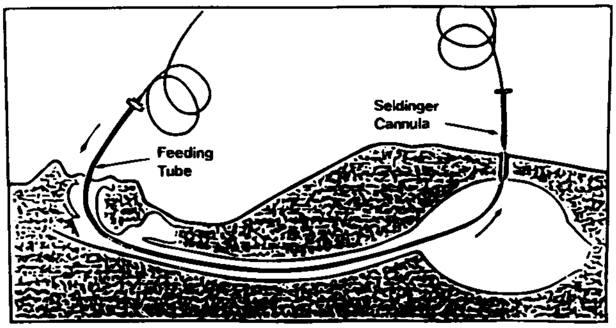

**Figure 2** Schema of the procedure of PEG.

into the skin incision, then through the abdominal wall into the stomach (Fig. 3c). When the endoscopist could see the Seldinger needle in the stomach, the polypectomy snear was looped loosely over the outer cannula (Fig. 3d). The soft guide wire was inserted through the Seldinger needle. When the guide wire was visualized in the stomach, slide the polypectomy snear down the outer cannula and snear the guide wire tightly. Both the endoscope and the polypectomy snear from the stomach and oropharynx is withdrawn as the guide wire is being freely fed into the cannula. The tapered dilator catheter with the attached gastrostomy tube was threaded over the guide wire. In the stomach, the leading end of the dilator catheter will follow its track as it is pushed back through the anterior abdominal wall. The leading end of the dilator catheter was grasped. As the tapered dilator was being pulled through the anterior abdominal wall, the bumper end of the gastrostomy tube was delivered safely through the oropharynx. The tube was gently pulled into position (Fig. 3e). Under endoscopic visualization, the gastrostomy tube bumper was pulled snugly up against the gastric mucosa. The guide wire was removed through the abdominal site. The proper bumper position was confirmed by endoscopy (Fig. 3f). The retention disc was passed over the gastric tube and secured. After placement of the gastrostomy tube into the patient, endoscopy must be performed to verify proper positioning of the bumper against the gastric mucosa. Although it is possible to feed the patient immediately after this procedure, it is good to wait until the

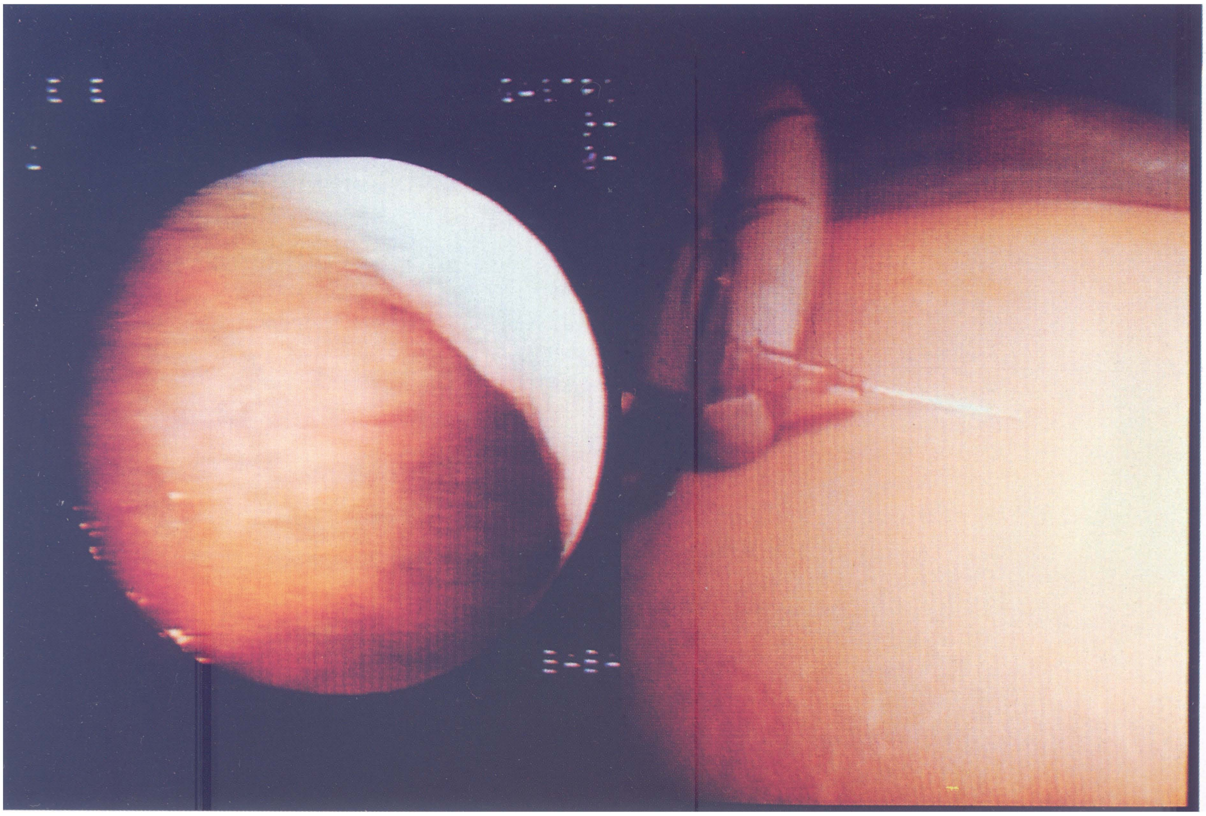

**Figure 3** Procedure. a. This endoscopic photograph demonstrates the puncture site of the stomach. b. Local anesthesia is made to the skin of puncture site.

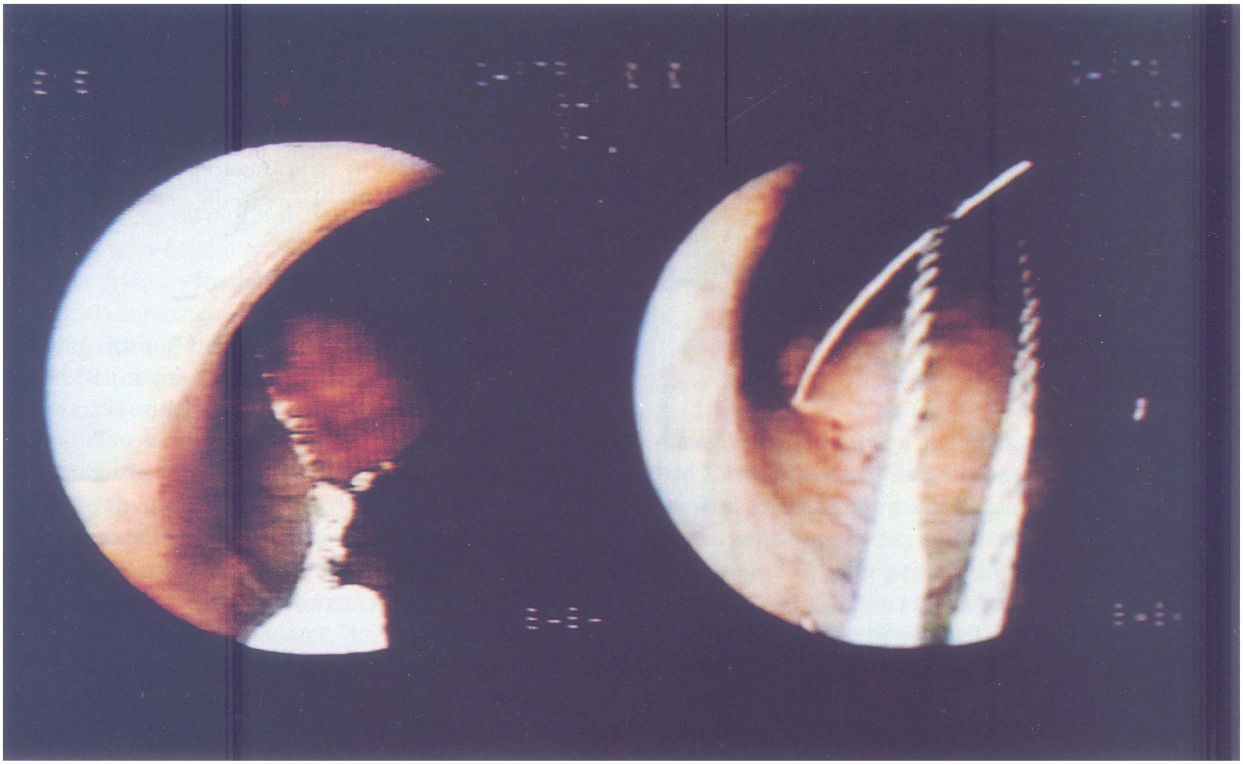

**Figure 3** Procedure. c. This endoscopic photograph demonstrates the polypectomy snare put on the puncture site. d. This endoscopic photograph demonstrates the guide wire grasped with snare.

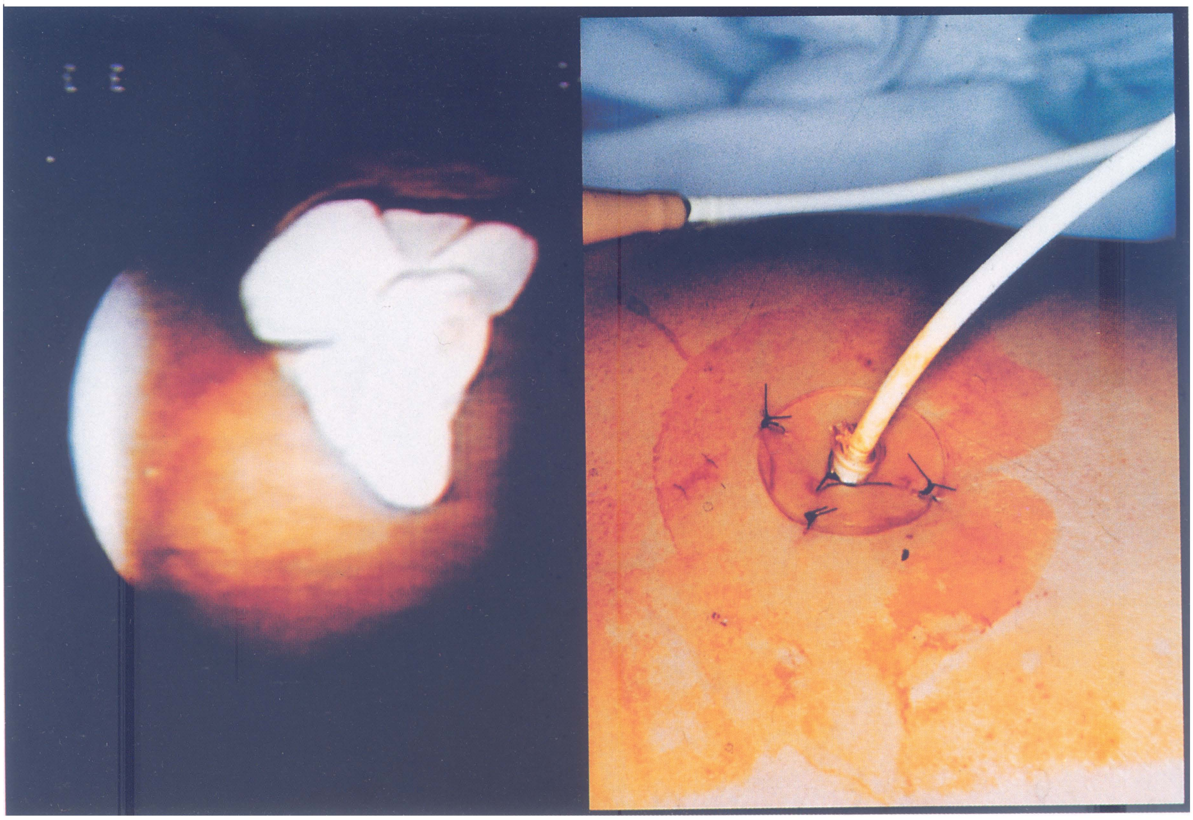

**Figure 3** Procedure. e. This endoscopic photograph demonstrates the bumper of the gastrostomy tube. f. Feeding tube is fixed with retension disc to the skin.

following morning before starting a liquid diet. Before starting meals, we usually confirm that there was no leakage of the contact media from the gastrostomy tube the next morning (Fig. 4). For the patients who had previously received gastrectomy, we carefully decided upon the puncture site using contact media. Reconstruction was by the Billroth I or Billroth II method, or by antecolica or retrocolica.

### RESULTS (TABLE 1)

A total of 34 patients ranging in age from 32 to 79 years have undergone this procedure. Almost all patients had neurological impairment following cerebral bleeding, infarction, or tumor. Some of the patients had respiratory impairment caused by the feeding tube, with nasal alimentation. They recovered their respiratory function soon after the placement of PEG. Of the 34 patients, there were no major complications. There were 4 minor complications such as peristomal infections, all of which were successfully treated with local drainage and antibiotics.

We attempted this procedure for the patient who had previously received gastrectomy. We carefully chose the puncture site, and we used the same procedure for these patients. They also had no major or minor complications. For these patients, we carefully decided the puncture site using contact media. We had 4 such cases and we achieved successful results without any trouble (Figs. 5 and 6).

### DISCUSSION

Gauderer and Ponsky (Gauderer and Ponsky, 1980) performed the first incisionless gastrostomy in 1979 using a homemade kit. Today, PEG which can be accomplished with local anesthesia and without the necessity for laparotomy has become popular in clinical treatment for these patients, and subsequent modifications of the technique of PEG has been accompanied by the production of commercial kits. The technique is widely practiced and reported in North America, but there have been few reports in Asia.

Although nasogastric tube feeding is simple to initiate and is less invasive than PEG, there are some disadvantages associated with its use, such as esophageal ulcer, and aspiration pneumonia. Complications are more likely to occur, the longer the tubes are left in place. Therefore, gas-

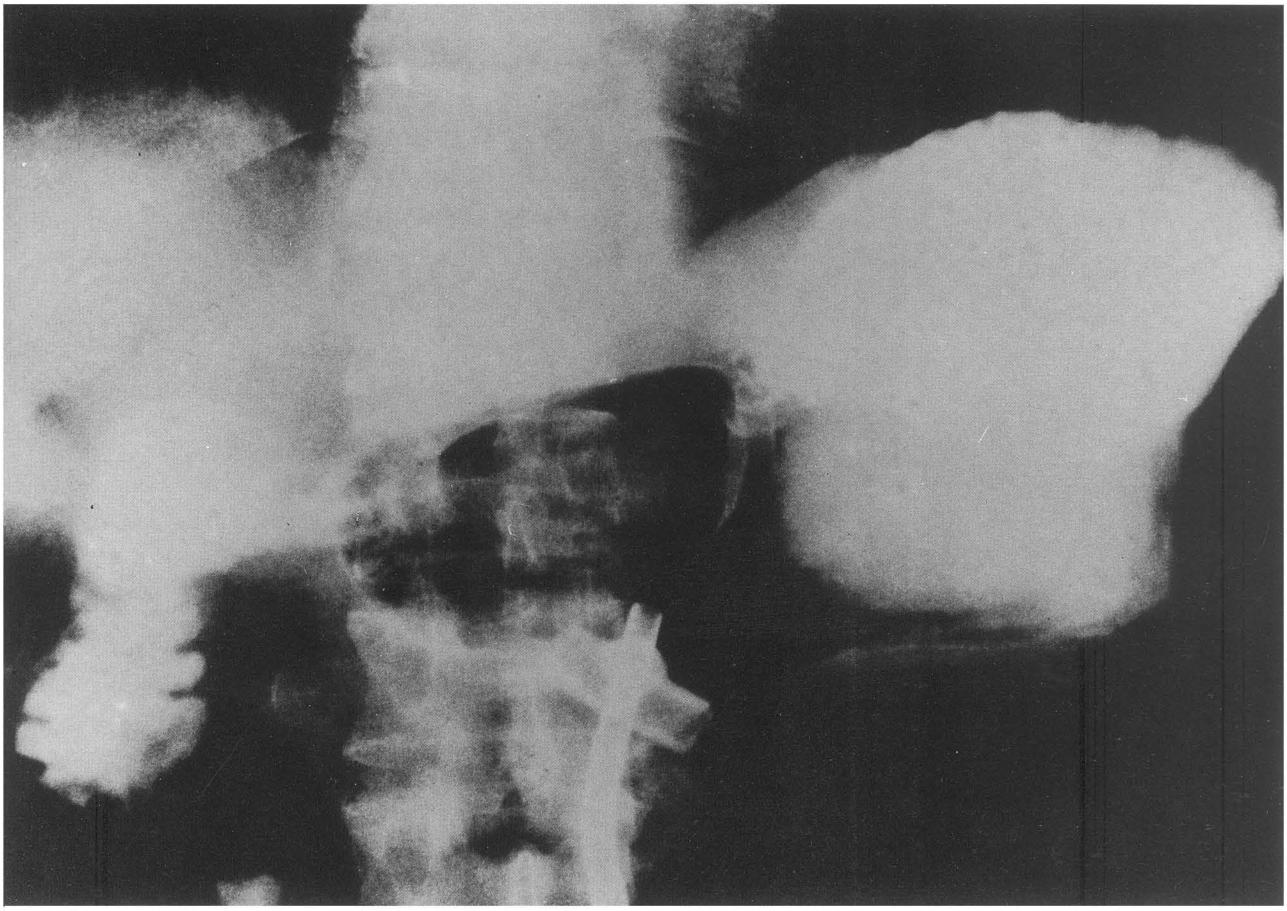

**Figure 4** Radiograph after PEG. No leakage of the contact media from the gastrostomy tube is found.

trostomy is a well recognized and satisfactory method of long-term enteral nutrition. PEG is best recommended for patients who have neurological disorders such as motor neuron diseases (bulbular palsy), brain injury (trauma or surgical), other neurological causes of difficulties in swallowing (Moran *et al.*, 1990).

Recent series report a failure rate of about 5% (Kirby *et al.*, 1986; Ponsky and Gauderer, 1989; Foutch *et al.*, 1988). The usual reason for failure is the inability to appose the stomach to the anterior abdominal wall because of previous surgery or morbidity obesity. Major complications such as aspiration, gastric perforation, colonic perforation, and gastric bleeding involving this procedure is reported from 3 to 5% (Moran *et al.*, 1990; Kirby *et al.*, 1986, Ponsky and Gauderer 1989, Foutch *et al.*, 1988; Ponsky and Gauderer 1981; Mamel 1988; Sangster *et al.*, 1988).

On the other hand, the incidence rate of minor complication, such as wound infection, pneumoperitoneum, etc. is reported to be from 4 to 13%. Wound infection is the most common minor complication. Jain *et al.* (Jain *et al.*, 1988) reported that administration of cephalosporin reduces peristomal wound infection from 28.6 to 7.4%. It is also recommended that the skin exit site should be slightly larger than the gastrostomy tube to allow any wound fluid or secretions to drain out (Foutch *et al.*, 1986). We had 4 out of 34 cases (12%) of wound infection but they were successfully treated and they were soon discharged.

Gastroesophageal reflux can be the problem and may lead to aspiration pneumonia. Reflux can be reduced by duodenal intubation. Modern kits are designed so that a smaller tube may be passed through the gastrostomy tube. For some patients who had recurrent aspiration pneumonia involving nasogastric tube feeding, it subsided soon after this procedure. Some investigators reported the comparison of percutaneous endoscopic gastrostomy with surgical gastrostomy. Gupta *et al.* (Gupta *et al.*, 1989) compared the insertion time, time from gastrostomy to initiation of feeding and major and minor complication rate

**Table 1** Patients Treated with PEG

| | *Age* | *Sex* | *Neurological disorder* | *Procedure* | *Surgical history* | *Complication* |
|---|---|---|---|---|---|---|
| 1 | 60 | F | CB | PEG | — | — |
| 2 | 60 | F | CI | PEG | — | — |
| 3 | 45 | M | CB | PEG | — | — |
| 4 | 63 | M | CB | PEG | — | — |
| 5 | 52 | M | CB | PEG | — | Subcutaneous abscess |
| 6 | 61 | M | CB | PEG | — | — |
| 7 | 60 | M | CB | PEG | — | — |
| 8 | 73 | M | CB | PEG | — | — |
| 9 | 60 | M | CI | PEG | — | — |
| 10 | 60 | M | CI | PEG | — | — |
| 11 | 43 | M | CI | PEJ | gastrectomy (B II) | — |
| 12 | 77 | M | CB | PED | gastrectomy (B I) | — |
| 13 | 56 | M | CB | PEG | gastrectomy (B II) | — |
| 14 | 44 | F | Guillan-Barre syndrome | PEG | — | — |
| 15 | 83 | M | CB | PEG | — | — |
| 16 | 33 | F | —*1 | PEJ | esphagectomy*3 | — |
| 17 | 79 | M | CI | PEG | — | — |
| 18 | 47 | F | CB | PEG | — | — |
| 19 | 77 | M | —*2 | PEG | — | — |
| 20 | 54 | M | CB | PEG | — | Subcutaneous abscess |
| 21 | 69 | F | CI | PEG | — | — |
| 22 | 58 | M | cord injury | PEG | — | — |
| 23 | 32 | M | CI | PEG | — | — |
| 24 | 67 | M | CI | PEG | — | — |
| 25 | 39 | M | CI | PEG | — | Subcutaneous abscess |
| 26 | 60 | M | CB | PEG | — | Subcutaneous abscess |
| 27 | 61 | F | CI | PEG | — | — |
| 28 | 67 | M | CB | PEG | — | — |
| 29 | 30 | M | CT | PEG | — | — |
| 30 | 52 | M | CI | PEG | — | — |
| 31 | 58 | F | CT | PEG | — | — |
| 32 | 22 | M | CT | PEG | — | — |
| 33 | 59 | F | CT | PEG | — | — |
| 34 | 68 | M | CT | PEG | — | — |

CI: Cerebral Infection; CB: Cerebral bleeding; CT: Cerebral Tumor; *1: oropharyngeal disorder; *2: aspiration pneumonia; B I: Billroth I; B II: Billroth II; *3: esophago-jejunostomy.

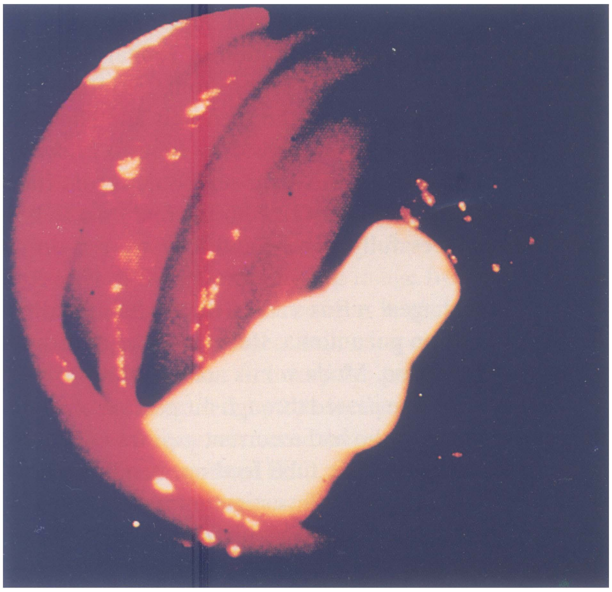

**Figure 5** This endoscopic photograph demonstrates the narrow lumen of duodenum in PED case.

in 50 percutaneous gastrostomies and 60 surgical gastrostomies performed during the same period. The two groups of patients were similar. Insertion time and time to initiation of feeding were significantly less in the percutaneous group. Procedure-related mortality rate was 2% in the percutaneous group compared with 7% in the surgical group. Major and minor complication rates were 2 and 12%, respectively after surgery. For the patients who had previously received gastrectomy, we carefully decided the puncture site using contact media. For these patients it is important to make sure whether reconstruction was Billroth I's method or Billroth II's method, antecolica or retrocolica. And it is more important to make sure that there are no other bowels between the abdominal wall and the bowel that is supposed to be punctured. Furthermore, the lumen of small intestine is narrow compared with the stomach, so we must be very careful on puncture (Fig. 5). We had 4 such cases and we could get successful results without any trouble (Fig. 6).

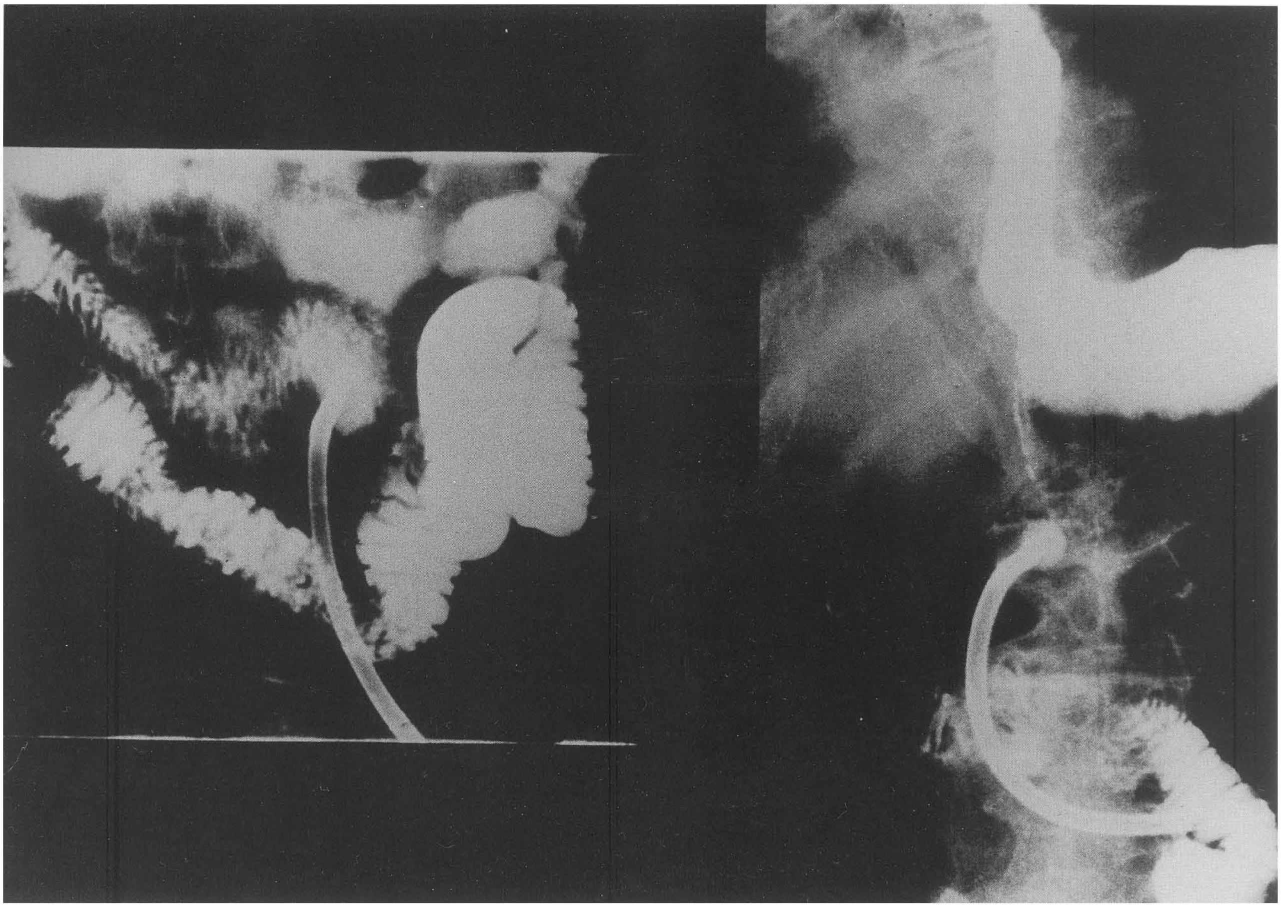

**Figure 6** Radiograph after PEJ (left) and PED (right). No leakage of the contact media from the feeding tube is found.

In conclusion, our results suggest that PEG is the method of safety for the patient, and is easy and fast to perform without the necessity of laparotomy.

### REFERENCES

Foutch P. G., Haynes W. C., Bellapravalu S.: Percutaneous endoscopic Gastrostomy (PEG): a new procedure comes of age. *J clin gastroenterol* 1986;8:10–15

Foutch P. G., Woods C. A., Sanowski R. A.: A critical analysis of the Sacks-Vine gastrostomy tube: a review of 120 consecutive procedures. *Am J Gastroenterol* 1988;83:812–815

Gauderer M. W. L., Ponsky J. L., Izant R. J.: Gastrostomy without laparotomy: a percutaneous endoscopic technique for feeding gastrostomy. *J Pediatr Surg* 1980;15:872–875

Gupta T., Maliakal B. J., Pelemn R., Ehrinpreis M., Weaver D., Luk G. D.: Complications of surgical (SG) and percutaneous endoscopic gastrostomy (PEG). *Gastroenterology* 1989;96:A190

Jain N. K., Larson D. E., Schroeder K. W.: Antibiotic prophylaxis for percutaneous endoscopic gastrostomy: a prospective, randomized, double blind clinical trial. *Ann Int Med* 1987;107:824–828

Kirby D. F., Craig R. M., Tsang T., Plotnick B. H.: Percutaneous endoscopic gastrostomy: a prospective evaluation and review of the literature. *JPEN* 1986;10:155–159

Mamel J. J. Percutaneous endoscopic gastrostomy. *Am J Gastroenterol* 1989;84:703–710

Moran B. J., Taylor M. B., Johnson C. D.: Percutaneous endoscopic gastrostomy. *Br J Surg* 1990;77:858–862

Okano H., Kodama T., Sato T. *et al.*: Percutaneous endoscopic gastrostomy = PEG. *Gastroenterological Endoscopy* 1986; 28:2114–2116. (in Japanese).

Ponsky J. L., Gauderer M. W. L.: Percutaneous endoscopic gastrostomy a nonoperative technique for feeding gastrostomy. *Gastrointestinal Endoscopy* 1981;1:9–11

Ponsky J. L., Guaderer M. W. L.: Percutaneous endoscopic gastrostomy: indications, limitations, techniques and results. *World J Surg* 1989;13:165–170

Sangster W., Cuddington G. D., Bachulis B. L. Percutaneous endoscopic Gastrostomy. *Am J Surgery* 1988;155:677–679

Yamada T., Ohnishi H., Matsuura T. *et al.*: Percutaneous endoscopic gastrostomy for elderly patients; A comparative study with nasogastric Feeding. *Dig Endosc* 1991;3:206–212

